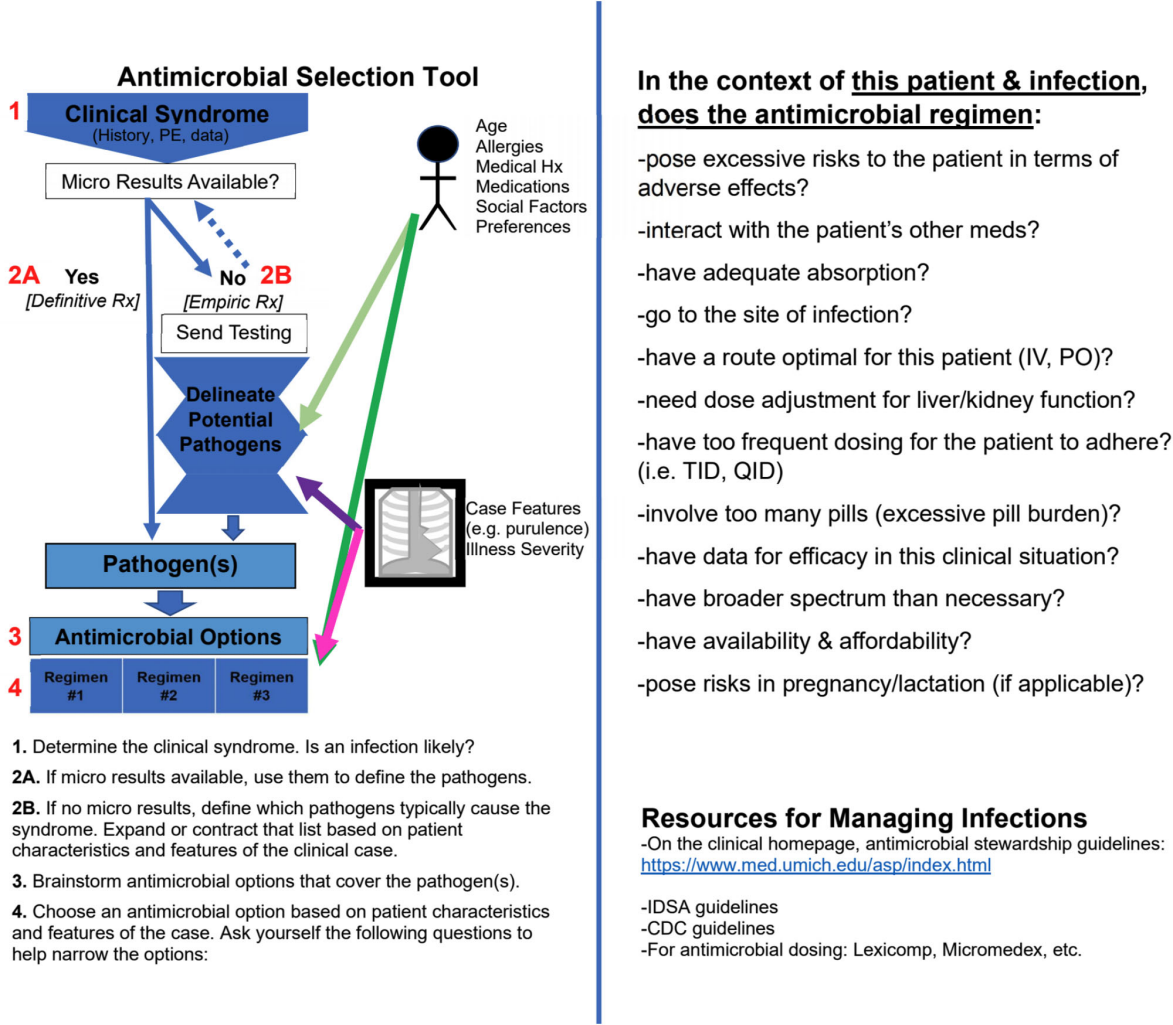# Teaching Antimicrobial Decision-Making in Medical Education: A Qualitative Study

**DOI:** 10.1017/ash.2024.153

**Published:** 2024-09-16

**Authors:** Elizabeth Scruggs-Wodkowski, Emily Abdoler

**Affiliations:** University of Michigan

## Abstract

**Background:** Inappropriate use of antimicrobials contributes to the growing threat of antibiotic resistance. While physicians encounter infections in virtually every facet of medical practice, research has shown that physicians have difficulty determining the need for antimicrobials and choosing the right drug. Physicians’ difficulties with antimicrobial prescribing likely begin early in medical education, yet little is known about how medical students learn to make antimicrobial choices. Our study sought to better understand how medical students learn antimicrobial decision-making, including the impact of a new learning tool introduced in the Infectious Diseases (ID) and Microbiology preclinical course. **Method:** From 2021-2023, we conducted 18 individual interviews with a purposive sample of medical students at the University of Michigan who had taken the preclinical ID/Microbiology course during the 2019-2021 curricular years. We asked participants how they learned to make antimicrobial decisions and how the course and clinical rotations influenced their understanding of antimicrobial choice. The six participants who took the 2021 course were additionally asked how an antimicrobial decision-making tool introduced that year impacted that process (Figure 1). The tool was adapted from prior work on antimicrobial reasoning (Abdoler et al, 2020). Participants were asked whether they remembered being introduced to the tool (approximately 18 months prior) and if they utilized it during their clinical rotations. Results were analyzed using Dedoose Software to facilitate thematic analysis. **Result:** Several themes emerged on analysis. Nearly all participants reflected that they learned elements of antimicrobial decision-making during clinical rotations, through observation or direct interaction with physician mentors and patients. Several participants described the preclinical period as content learning, with clinical rotations providing a space to consolidate and scaffold knowledge, as well as transfer knowledge to new situations or tasks. Of the 6 students interviewed regarding the antimicrobial decision-making tool, only one remembered it and could accurately describe its components prior to being shown the tool during the interview. **Conclusion:** Results suggest that participants view the preclinical ID/Microbiology course primarily as an opportunity to learn content, and perceive learning antimicrobial decision-making directly from practicing physicians in the clinical portion of medical school. An antimicrobial decision-making tool introduced during the preclinical ID/Microbiology course in 2021 did not impact students’ conceptualization of how they learned this skill. Given that practicing physicians often make antimicrobial prescribing errors, regular re-introduction of the tool during clinical rotations may help bridge preclinical antimicrobial educational content to the clinical phase of learning, counteract inappropriate antimicrobial lessons encountered clinically, and ground students' burgeoning antimicrobial prescribing skills in a logical reasoning model.

**Figure:**